# Microscopical Examination of Human Blood in a Case of Leukæmia Reported by MacGillavry

**Published:** 1881-06

**Authors:** P. W. Van Peyma


### Translations.

---

### MICROSCOPICAL EXAMINATION OF HUMAN BLOOD IN A CASE OF LEUKÆMIA. REPORTED BY MAC GILLAVRY.

FROM THE DUTCH BY P. W. VAN PEYMA, M. D.

A few days since my friend, Mr. P. Korteweg, informed me that at the polyclinic of Prof. Rosenstein a case of leukæmia with considerable enlargement of the spleen had been observed. He added that in the blood of this patient white blood-corpuscles of giant size had been demonstrated. Prof. Rosenstein was absent from Leyden, and his return not expected for a number of days. At my request, Mr. Korteweg furnished me on the last day of the preceding year with a drop of blood obtained from the patient in question, who at the time was an inmate of the hospital. In company with Messrs. Korteweg and Goddard, assistant to Prof. Rosenstein, I without delay, proceeded to examine the preparation furnished me.

The number of colorless corpuscles therein contained was wonderfully great. What was, however, more remarkable was the variation in the size of the objects contained within the field of vision: side by side with normal colored blood-corpuscles, that in many instances had formed into so-called rolls of coin, there were to be seen bodies exceedingly minute, apparently similar in character both chemically and physically (microcyts ?).

The colorless bodies presented great variations in size, but, as compared with the normal corpuscles, were exceedingly small. The majority possessed cyst-like nuclei; others were probably fragments of broken-down leucocytes, of course wanting in nuclei; and lastly, very large protoplasmic bodies in which no nuclei were discernible. Forms transitional between the normal and the very large were also numerous.

While I was examining the preparation with one of Zeiss' admirable lenses (F. with correction) and a No. 1 eye-piece, the diaphragmatic opening having been made as small as possible, there were observed contained within the leucocytes a number of granules indistinctly visible, equidistant from each other, and differing in appearance from those seen in the protoplasm of cells undergoing fatty degeneration. During the examination I first remarked that these granules resembled the micrococci in "zoogloic masses," soon following this with the assertion that they appeared in reality to be micrococci, and I thereupon determined to submit my views to the test of experiment.

A drop of aqueous solution of picro-carmine was carefully added, the excess being removed by means of absorbent paper. Immediately the outlining of the granules became sharper, and as I was calling the attention of Mr. Korteweg to the fact, the large leucocyte, in which the granules were contained, made some peculiar movements and burst. There remained a homogeneous mass which became rapidly colored red, while the granules, the former contents, underwent the common molecular movements described by Brown. I was therefore not fortunate enough to observe positively the movements well-known to me peculiar to bacteria. I notice here that amongst the micrococci set free, I observed one possessing the figure eight or dumb-bell shape. My idea that I was dealing with the micrococci of leucocytes became now more probable, and still more so when in due time I established:

1st, That the large homogeneous spherical masses, from which (following the addition of the aqueous solution of picrocarmine) the small granules had escaped, had become deeply red, while the similarly appearing colorless corpuscles remained entirely colorless; and,

2d, That the granules had through the coloring matter become colored yellowish-brown and so sharply defined in the field of vision, that even to the inexperienced they were apparent at a glance.

Early on the following day I sought to obtain another drop of the patient's blood, and as soon as this was done, I, with the assistance of Mr. Korteweg and Dr. Nykamp, resumed my work. Dr. Nykamp had the kindness to make three inoculations of the leukæmic human blood upon each ear of an apparently healthy rabbit. The blood used being diluted with a six per cent. solution of common salt. Around one of the ears a ligature was placed, with the object of diminishing the chances of the connective cells in their contest with the micrococci.

If one may reason from former inoculations of leukæmic human blood, then it is hardly probable that my rabbit will give positive results. But time will determine the matter. I will here simply say that the blood obtained from the rabbit at the time of the inoculation showed a very small number of white globules, but there were noticeable a number of psorosperms, aggregated in groups.

During this time I busied myself with the examination of the fresh blood. The addition of a five per cent. potassa solution failed to dissolve the granules. The picro-carmine reaction again gave abundant results. By means of a mixture of sodium chloride and picro-carmine, the exact proportions of which I do not know, I succeeded in protecting the large leucocyte from bursting. The protoplasm was again rapidly colored red and the contained micrococci yellowish-brown. As on the previous day, no nuclei were observable in the very large leucocytes.

For the benefit of the reader who has made few, if any, observations of micrococci, I take the liberty to mention that in the examination of micrococci one often finds minute granules greatly resembling the real micrococci. Here, perhaps, more than in the macrocosm is it true that there is more similarity than sameness. How are we to distinguish the real from the false micrococci?

1st. Observe the presence or absence of the characteristic movements.

2d. Notice the arrangement and size of the granules, whether they are equi-distant and of equal size.

3d. Diligently search for the rod-shape forms and with strong objectives to discover the dumb-bell or figure-eight forms.

4th. The addition of strong potash solution.

5th. Cause the absorption of solutions of coloring matters—for example picro-carmine, hæmatoxyline, etc.

From the above it follows that I cannot say positively to have seen the characteristic movements of the granules. If this had been the case, the granules would clearly have been properly classified. But this proposition can not be reversed, as many micrococci do not seem to posses the power of individual movement. Conclusion: reaction No. 1 leaves the matter doubtful.

The peculiar arrangement and the quite regular position of the granules first led me to the opinion that I had before me parasites of the white blood-corpuscles. Conclusion: reaction No. 2 leaves the proposition not improbable.

The dumb-bell-shaped forms were positively observed. Conclusion: reaction No. 3 increases the probability of the proposition.

Potassa solution does not effect the granules. Conclusion: the probability is further increased.

Picro-carmine effects all that is required by the hypothesis; normal leucocytes do not change or become colored; the larger possessing nuclei and a few granules also fail to become colored; the very largest no longer containing nuclei, but full of granules, are almost immediately colored, the protoplasm being here already dead; the coloring matter here no longer brings out the nuclei, which means simply that they no longer exist. This gives an additional proof of the death of the leucocyte. The granules themselves become yellowish-brown. Conclusion: the granules are micrococci.

Everything considered, I feel warranted in concluding that in a certain case of well-developed leukæmia the white blood-corpuscles were not only increased in number, and in part increased in size, but that the blood-corpuscles served as habitation for micrococci, and that the death of the leucocytes was due to the contained or inhabiting parasites. From the fact of the rarity of cases of leukæmia, I have felt called upon to publish to my professional brothers my experience as detailed. Possibly some may have cases throwing light upon the morbid process or processes still, alas, so dark, which we term leukæmia. Possibly also my observations may serve to lighten the work of others. * * *

If it should in the future be shown that the case in point is not one of simple leukæmia, but a morbid process hitherto not described, then I propose to give it the name of leucomycosis.—*Nederlandsch Tysdchrift voor Geneeskunde.*

